# Disabling foot pain and its impact on daily living among people with psoriatic arthritis in Singapore: a cross-sectional observational investigation

**DOI:** 10.1186/s41927-024-00409-3

**Published:** 2024-10-09

**Authors:** Vanessa H. Y. Teo, Kai Li Chia, Catherine Bowen, Manjari Lahiri, Peter P. M. Cheung, Deborah E. Turner, Kate Carter

**Affiliations:** 1 School of Health Sciences, Faculty of Environmental and Life Sciences, University of Southampton, Southampton, SO17 1BJ UK; 2 Division of Rheumatology, Department of Medicine, National University Hospital, Singapore, 119074 Singapore; 3 Department of Medicine, Yong Loo Lin School of Medicine, National University of Singapore, Singapore, Singapore; 4 Faculty of Health, School of Clinical Sciences, Queensland University of Technology, Podiatry, Brisbane, 4059 Australia; 5 School of Allied Health, Podiatric Medicine and Surgery Discipline, The University of Western Australia, Perth, 6009 Australia

**Keywords:** Psoriatic arthritis, Multidisciplinary team care, Disabling foot pain, Disease impact, Singapore

## Abstract

**Background:**

Psoriatic Arthritis (PsA)-related foot involvement has been shown to have a profound impact on daily functioning, with most studies having focused on predominantly Caucasian populations. The aim was to describe disabling foot pain (DFP) and its impact on daily living in PsA in Singapore.

**Methods:**

A cross-sectional, retrospective study was conducted using clinical data collected during a single-visit to a rheumatology clinic in Singapore. Records for adults with physician-diagnosed PsA were reviewed for sociodemographic information, disease characteristics, global disease activity and burden. Foot-specific measures included clinical assessment and the Manchester Foot Pain and Disability Index used to define DFP and evaluate between-group differences.

**Results:**

Forty-two participants with PsA (83% female, 57% Chinese, 31% Malay, 9.5% Indian, mean (SD) age 54-years (16)) attended the rheumatology clinic over the study-period. The median (IQR) disease duration was 2-years (11) and all were taking current DMARDs. Global disease measures demonstrated mild-to-moderate global disease activity and mild functional impairment, and were significantly higher in those with DFP. Despite 90% reporting to be coping well with their condition, self-care and having emotional support (*n* = 38), this study sample demonstrated high levels of anxiety/depression (29%), sleep disturbance (34%) and fatigue (24%), and a lack of disease- and drug-specific knowledge (64%). Further management was indicated for medication adherence counselling (48%), occupational therapy (43%), physiotherapy (36%) and podiatry (30%). Nearly half had current foot pain with 40% reporting DFP (*n* = 17), which caused significantly greater difficulty walking 3 km than those without DFP (*p* < 0.05). Rearfoot enthesitis (plantar fasciitis, Achilles enthesitis) was the most common cause of DFP (67%) with pain lasting longer than 1-year. 72% were overweight or obese, with a high proportion not engaging in any cardiovascular exercise (70%). Three of 42 participants had previously seen a podiatrist.

**Conclusions:**

People with DFP in PsA experience more severe global disease activity, reduced mobility and higher levels of negative impact on their daily lives in Singapore. In the absence of working in a multidisciplinary-team, there is value in comprehensive assessments that have potential to capture a holistic view of personal impact and improve person-centred care in PsA.

## Background

Psoriatic arthritis (PsA) is a chronic inflammatory disease with heterogeneous musculoskeletal and dermatological manifestations [1], and is characterised by disease features such as peripheral arthritis, dactylitis, enthesitis and psoriatic skin and nail disease [1, 2]. Global prevalence and incidence of PsA are estimated to be 133 in every 100,000 persons, and 83 in every 100,000 person-years respectively [3]. However, true global prevalence is difficult to determine due to the diverse expression of disease and the historical differences in classification criteria applied [4, 5].

PsA is associated with foot and ankle pain, structural changes leading to joint deformity and impaired function, which can have a wide-reaching impact on daily life [6]. Foot pain has been shown to be both predominant and persistent among people with PsA [7, 8], with consequent changes in gait parameters such as a slower walking speed as a potential unconscious mechanism by which to stress-shield entheseal structures and reduce plantar pressures on inflamed joints [9, 10]. Foot pain and these foot-related structural and functional impairments in PsA have been shown to negatively impact on daily routine, limit family and social activities, as well as lead to changes in job roles and work status [6, 11].

Despite the high prevalence of foot involvement in PsA, its impact on the daily lives of people with PsA remains under-researched with most studies from the UK, Europe and Australia having focused on predominantly Caucasian populations [6, 10, 12–14]. Limited research data suggests that the clinical presentation of PsA in Asian populations is known to be different compared with Caucasians [15, 16]. A few small PsA-specific studies have been conducted on Asian populations, revealing that differences in ethnicity, environmental factors and lifestyle may play a role in the prevalence and impact of PsA [15–19]. Significant variation in prevalence is seen across geographic locations and ethnic populations. For example; lower prevalence has been reported in Asian countries like Japan and China (0.1 in 100,000, and 2 in 100,000 respectively) [18]; PsA in Korea had predominant spinal involvement; Chinese may have a milder course in relation to impact on physical function; and Indians with psoriasis had twice the risk of developing PsA compared with Chinese [16, 19]. Compared with Chinese, Malay and European populations, ethnic South Asians may have greater disease activity and experience poorer physical function [15–17, 20]. This finding was also observed in South Asians living in both Western and Asian countries, suggesting a stronger influence from genetic rather than geographic factors [21]. Therefore, it follows that a better understanding of psoriatic foot disease in Singapore would facilitate foot health management service planning and may improve the patient experience and their outcomes.

Singapore is a multiethnic South-East Asian country with a majority ethnic Chinese population (74%), followed by Malay (14%) and Indian (9%) [22]. Variations in lifestyle and available social and healthcare support between countries may influence foot pain severity, foot-related disability and its consequent impact on daily life in a Singaporean PsA population. Disabling foot pain (DFP) is the experience of foot pain-related problems that can be assessed using the Manchester Foot Pain and Disability Index (MFPDI) across 4 constructs: pain intensity; functional limitations; personal appearances; and limitations in work and leisure activities [23, 24]. DFP has been associated with reduced functional ability in the general population including self-care [25, 26], increased risk of falls [27], depression [28] and reduced physical and mental aspects of quality of life [29], and is more likely to occur in people previously diagnosed with inflammatory arthritis [28, 30]. Determining the level of DFP in an outpatient population in Singapore would help to facilitate a better understanding of the burden of foot pain in the context of global disease activity in PsA. The aim of the study was to evaluate DFP and its impact on daily living among people with PsA in Singapore.

## Methods

### Study design

This investigation followed a cross-sectional, retrospective study design in which secondary analysis of a primary data set was conducted. Records were reviewed for clinical data that had already been collected as part of routine clinical care and an existing larger study [31] at the One-Stop Arthritis Clinic (OSAC) at the National University Hospital Rheumatology department in Singapore. The data collected included clinical examination, patient-reported outcomes using face-to-face and self-report questionnaires, and review of medical records as part of standard clinical practice (DSRB Reference: 2022-00037, Research Collaboration Agreement Reference: RITM0450258).

### Setting

In 2016, the OSAC was set up as a clinical practice improvement initiative to improve access to allied-health services for people with inflammatory arthritis who may benefit from holistic care. Prior to the OSAC, multidisciplinary team (MDT) care for people with inflammatory arthritis was sporadic, if at all. Low uptake from patients of allied-health appointments was attributed to a lack of awareness of the role of allied-health professionals with it perceived as unnecessary, the inconvenience of a separate visit, having a doctor-centered view of healthcare delivery, additional out-of-pocket cost, and low levels of health literacy among older patients in Singapore [32]. Therefore, the OSAC was established with the aim of providing point of care access to MDT care. The OSAC operated as a single visit to a 6-member MDT clinic, which comprised the rheumatologist, rheumatology specialist nurse, podiatrist, physiotherapist (PT), occupational therapist (OT) and medical social worker (MSW). Existing patients on follow-up at the rheumatology outpatient clinic may be referred, by discretion of their rheumatologist, to the OSAC, where consenting patients were seen there in lieu of their routine rheumatology review. At the OSAC all members of the MDT were co-located in a single clinic, and every MDT member assessed each patient with the capacity of 6 patients per clinic session.

### Participants

Adults (≥ 21 years) with physician diagnosed PsA who attended a single visit to the multidisciplinary rheumatology outpatient clinic were included for data analysis. Participants with other forms of inflammatory arthritis were excluded. Data for this investigation was reviewed over a period from April 2016 to April 2022, with the clinic having operated on a once-a-month basis (2016–2019) and subsequently a quarterly basis (2022), and the clinic was not operating due to the Covid-19 pandemic between 2020 and 21 and part of 2022.

### Data collection

Sociodemographic characteristics included age, gender, ethnicity, marital status, education level, primary language spoken, occupation and work status. Clinical characteristics including disease duration, body mass index, current pharmacological management and the presence of comorbidities were recorded.

Global disease activity measures included the following:

- Patient Global Assessment [33] and global pain [34] using an 11-point numerical rating scale (NRS), the Physician Global Assessment [35] using a 100 mm visual analogue scale (VAS) with higher scores indicating worse pain or worse global health;
- Routine Assessment of Patient Index Data-3 (RAPID3) was used to assess the severity of global disease activity. Validated for use in PsA [36], the RAPID3 is a pooled index of 3 patient-reported measures including physical function (scored 0–10), pain (0–10) and patient global estimate of status (0–10), with a total score of 0 to 30 where > 12 indicates high disease activity, 6.1 to 12 as moderate, 3.1 to 6 as low, and ≤ 3 as remission [37];
- Tender (TJC-68) and Swollen Joint Counts (SJC-66) were performed by the rheumatologist [38];
- Radiographic findings were the presence of bony erosions in the hands and feet [39];
- Erythrocyte sedimentation rate (ESR, mm/hr) and C-reactive protein (CRP, mg/L) [40, 41];
- Multi-dimensional Health Assessment Questionnaire (MD-HAQ) measured physical function [39, 42] and the original HAQ has been shown to be reliable for use in PsA [39]. The MD-HAQ consists of 10 items that assess the extent of difficulty with daily activities and is rated on a 4-point Likert scale (0 = without any difficulty, to 4 = unable to do so), with scores ranging from 0 to 3 where 0 to 1 indicates mild/moderate functional impairment, 1.1 to 2 being moderate/severe and 2.1 to 3 being severe/very severe impairment [43].
- European-QoL 5-dimensional level-3 questionnaire (EQ-5D-3 L) measured health-related quality of life and has shown discrimination and responsiveness in PsA clinical trials [39]. The EQ-5D-3 L measures 5 dimensions: (1) mobility, (2) self-care, (3) usual activities, (4) pain/discomfort and (5) anxiety/depression, which are evaluated from no problem to extreme problem and scored from 1 to 3. The 100 mm VAS component of the EQ-5D-3 L was used to assess the overall health status and is rated from 0 being worst imaginable health status to 100 being best imaginable health status [39].

Foot and ankle characteristics included foot pain, foot deformity, foot related-functional impairment and disability, and PsA disease features. The MFPDI was used to define DFP [23]. The MFPDI is a patient-reported outcome measure that comprises 19-items that assess 4 constructs: pain intensity (5 items), functional limitations (10 items), personal appearances (2 items), and limitations in work and leisure activities (2 items). The scores are totaled for a maximum of 38, with response options as ‘none of the time’ = 0, ‘on some days’ = 1, and ‘on most days/every day’ = 2 [23]. Participants with at least 1 of the 10 functional limitation items documented on most/every day(s) were those classified as having DFP – a definition that has been shown to have good internal consistency and substantial repeatability [24]. Participants were grouped into those with and without DFP in order to investigate between-group differences.

Forefoot and rearfoot deformities were quantified using the Structural Index (SI) score [44], those with a forefoot SI score of *≥* 10 or a rearfoot SI score of *≥* 4 indicated the presence of severe foot deformity [45]. Experience of previous and current foot pain as well as having been previously referred to and seen by a podiatrist was noted. Current foot pain severity was measured using a 100 mm VAS with 0 as no pain and 100 as worst ever pain for the question ‘How severe is your foot pain?’ PsA disease features in the foot that had been recorded by clinical foot examination by the podiatrist were included.

### Data analysis

The data analysis strategy was planned a priori with self-reported measures having been selected from the primary data set for analysis. Domains selected for data analysis were based on recommendations made by the 2016 Group for Research and Assessment of Psoriasis and Psoriatic Arthritis (GRAPPA) and Outcome Measures in Rheumatology (OMERACT) PsA core domain set [46]. The disease domains and the scale items selected were directly relevant to the domains of impact on daily life, participation and coping as well as disease metrics that had shown reliability for use in PsA [39]. The outcome measures and clinical assessment items selected were:

1. **Disease activity** (RAPID3).
2. **Physical function** in mobility and walking ability (MD-HAQ, EQ-5D-3 L).
3. **Participation** in recreational activities and sports (MD-HAQ), activities of daily living and work (MFPDI), types of leisure activities (OT assessment), and engagement in cardiovascular exercise (PT assessment).
4. **Emotional well-being** included feelings of anxiety and depression (MD-HAQ, EQ-5D-3 L), ability to cope with self-care, domestic tasks and leisure activities (OT assessment), ability to cope with their condition and types of coping strategies (MSW assessment), access to social and emotional support (MSW assessment), understanding of their disease and medications (nurse assessment), and further healthcare support indicated (OT, PT, MSW, nurse and podiatry assessment).
5. **Sleep** (MD-HAQ).
6. **Fatigue** (OT assessment).

Demographic, clinical and foot characteristics are presented as means and standard deviations (SD). For data not normally distributed, median and interquartile range (IQR) are used, and categorical data are presented as numbers and percentages. Descriptive statistics for global disease measures and indices were generated for those with and without DFP, and mean differences between groups were analysed using the 2-tailed Independent T test and 2-tailed Mann-Whitney U test for parametric and non-parametric variables respectively. For the selected domains of disease impact, Fisher’s exact test was used to determine statistical significance between groups. All statistical tests were conducted at a 5% level of significance and were conducted using the Statistical Package for Social Sciences (SPSS) v28.0.1.1 (Inc. Chicago. Illinois). Missing data refers to an unrecorded data value, which has been identified for each variable and reported in the study tables. Missing data was below 5%, which is regarded inconsequential for non-biasing results and can be reported using descriptive analysis and not imputation techniques [47, 48].

## Results

Key study findings are summarised in Tables 1, 2, 3 and 4. Analysis was conducted on a total of 42 participants with PsA who had attended the OSAC rheumatology clinic during the defined study period. The majority of participants were female (83%), Chinese (57%), with a mean (SD) age of 54-years (16). The median (IQR) disease duration was 2-years (11) and all were taking current disease modifying anti-rheumatic drugs (DMARDs). Most were married (81%) and lived with family members (95%), were not in paid employment (43%), and had an education at Secondary level and beyond (74%). Housewife/domestic work was the most common occupation (33%).

**Table 1:** Sociodemographic and clinical characteristics of participants with and without disabling foot pain (DFP) in PsA

	Total *n* = 42	with DFP *n* = 17	without DFP *n* = 25
Female (n, %)	35 (83)	16 (94)	19 (76)
Age (years) (mean, SD)	54 (16)	52 (16)	55 (15)
Ethnicity (n, %)			
Chinese Malay Indian Others	24 (57) 13 (31) 4 (10) 1 (2)	9 (53) 4 (23) 3 (18) 1 (6)	15 (60) 9 (36) 1 (4) 0 (0)
Body mass index (kg/m^2^) (mean, SD)			
Underweight (< 18.5)	0	0	0
Healthy weight (18.5–24.9)	12 (28)	5 (29)	7 (28)
Overweight (25-29.9)	20 (48)	9 (53)	11 (44)
Obese (> 30)	10 (24)	3 (18)	7 (28)
Marital status (n, %)			
Married	34 (81)	13 (76)	21 (84)
Single	5 (12)	2 (12)	3 (12)
Widowed	1 (2)	0 (0)	1 (4)
Divorced	2 (5)	2 (12)	0
Living arrangements^+^ (n, %)			
Alone	3 (7.5)	2 (12)	1 (4)
With Family	37 (92.5)	15 (88)	22 (96)
Education level (n, %)			
None	4 (10)	1 (6)	3 (12)
Primary	7 (17)	2 (12)	5 (20)
Secondary	18 (43)	10 (59)	8 (32)
Vocational	1 (2)	0 (0)	1 (4)
Diploma	6 (14)	1 (6)	5 (20)
Degree	6 (14)	3 (17)	3 (12)
Primary language (n, %)			
English	28 (66)	12 (71)	16 (64)
Mandarin	10 (24)	4 (23)	6 (24)
Chinese dialect	2 (5)	0 (0)	2 (8)
Malay	2 (5)	1 (6)	1 (4)
Occupation^+^ (n, %)			
Unemployed/retired	5 (12)	1 (6)	4 (16)
Housewife/domestic	13 (32)	4 (24)	9 (38)
Manual work	1 (3)	0 (0)	1 (4)
Sales/admin	10 (24)	7 (41)	3 (13)
Professional	2 (5)	0 (0)	2 (8)
Others	10 (24)	5 (29)	5 (21)
Work status (n, %)			
Full time	19 (45)	11 (65)	8 (32)
Part time	6 (14)	1 (6)	5 (20)
Not working/Retired	17 (41)	5 (29)	12 (48)
Comorbidities (n, %)			
Diabetes (Type II)	7 (17)	2 (13)	5 (18.5)
Disease duration (years)^+^ (median, IQR)	2 (11)	1 (12)	2 (9)
Less than 2 years (n, %)	17 (45)	9 (56)	8 (36)
2 years or more (n, %)	21 (55)	7 (44)	14 (64)
Hand Radiograph^+^ (n, %)			
Erosions	8 (30)	4 (36)	4 (25)
Joint space narrowing	14 (52)	7 (64)	7 (44)
Foot Radiograph^**+**^ (n, %)			
Erosions	5 (33)	4 (57)	1 (13)
Joint space narrowing	4 (27)	3 (43)	1 (13)
Medications^+^ (n, %)			
NSAID	18 (45)	10 (63)	8 (33)
Prednisolone	12 (34)	5 (36)	7 (33)
csDMARD	40 (100)	15 (100)	25 (100)
Biologic & csDMARD combined	2 (5)	1 (7)	1 (4)

^**+**^ Missing data

NSAID non-steroidal anti-inflammatory drugs, csDMARDs conventional synthetic disease modifying anti-rheumatic drugs, IQR interquartile range, SD standard deviation

**Table 2:** Global disease measures and disease indices of participants with and without disabling foot pain (DFP) in PsA

	Total *n* = 42	with DFP *n* = 17	without DFP *n* = 25	*p*-value
MD-HAQ (median, IQR)	0.3 (0.6)	0.5 (0.7)	0.2 (0.5)	0.03*
Global Pain (NRS 0–10) (median, IQR)	3 (4)	4 (2)	2 (4)	0.009*
Patient Global Assessment (NRS 0–10) (median, IQR)	3 (4)	5 (2)	2 (5)	0.006*
Physician Global Assessment (VAS 0–100 mm)^**+**^ (mean, SD)	26 (20)	36 (24)	21 (15)	0.048*
RAPID3 (mean, SD)	7.5 (5)	10.6 (4)	5.6 (5)	0.002*
VAS for general global health, part of the EQ-5D-3 L (0–100 mm) (mean, SD)	62.4 (16)	57.3 (14)	65.8 (27)	0.092
ESR^**+**^ (mm/hr) (median, IQR)	27.5 (25)	27.5 (25)	25 (25)	0.56
CRP^**+**^ (mg/L) (median, IQR)	8 (9)	6 (9)	9.5 (14)	0.78
SJC-66 (median, IQR)	0 (8)	8 (19)	0 (2)	0.03*
TJC-68 (median, IQR)	0.5 (9)	12 (19)	0 (2)	0.01*
SJC - foot and ankle (median, IQR)	0 (3)	2 (10)	0 (1)	0.006*
TJC - foot and ankle (median, IQR)	0 (4)	3 (11)	0 (1)	0.001*

* Significant *p* value found (*p* < 0.05), ^**+**^ missing data

MD-HAQ multi-dimensional Health Assessment Questionnaire, VAS visual analogue scale, NRS numerical rating scale, RAPID3 Routine Assessment of Patient Index Data 3, ESR erythrocyte sedimentation rate, CRP c-reactive protein, EQ-5D-3 L European-QoL 5-dimensional level-3 questionnaire, SJC-66 swollen joint count 66, TJC-68 tender joint count 68, IQR interquartile range, SD standard deviation

**Table 3:** Foot and ankle characteristics of participants with and without disabling foot pain (DFP) in PsA from podiatric clinical assessment

	Total *n* = 42	with DFP *n* = 17	without DFP *n* = 25
Past foot problems^+^ (n, %)	31 (76)	17 (100)	14 (58)
Current foot problems^+^ (n, %)	20 (49)	14 (82)	6 (25)
Previously been referred to and seen a podiatrist (n, %)	3 (7)	2 (12)	1 (4)
Current foot problem duration^+^ (> 1 year)	9 (45)	7 (58)	2 (25)
Location of foot problems^+^ (n, %)			
Forefoot	11 (55)	8 (57)	3 (50)
Midfoot	3 (15)	3 (21)	0 (0)
Rearfoot	10 (50)	8 (57)	2 (33)
Foot pain levels^+^ (VAS 0–100 mm) (mean, SD)	45 (24)	49 (21)	39 (29)
Disease features in the foot (n, %)			
Skin psoriasis on the foot	10 (24)	3 (18)	7 (26)
Psoriatic toenails	12 (29)	5 (29)	7 (28)
Dactylitis	7 (17)	5 (29)	2 (8)
IPJ arthritis	10 (24)	4 (24)	6 (24)
Tendinopathy	7 (17)	3 (18)	4 (16)
Enthesitis	17 (41)	11 (65)	6 (24)
Achilles tendon	6 (14)	5 (29)	1 (4)
Plantar fascia	8 (19)	5 (29)	3 (12)
Tibialis Posterior*	3 (7)	2 (12)	1 (4)
Peroneal*	6 (14)	3 (18)	3 (12)
Structural Index^+^ (median, IQR)			
Forefoot	2.0 (6.0)	4.0 (6.0)	1.0 (7.0)
Rearfoot	2.5 (5.0)	3.0 (6.0)	2.5 (6.0)
Total	7.0 (12.0)	7.0 (13.0)	5.5 (10.0)

^+^Missing data. *Comprising classic and functional enthesitis: Classic enthesitis at the insertion of the tibialis posterior tendon to the navicular and the peroneus brevis tendon to the proximal fifth metatarsal, and functional enthesitis at the sites where tendons wrap closely around bony pulleys (tibialis posterior tendon around the medial malleolus and peroneal tendons around the lateral malleolus)

VAS Visual analogue scale, IPJ Inter-phalangeal joint, IQR interquartile range

**Table 4:** The presence of disabling foot pain (DPF) and selected data aligning with the domains of disease impact defined by the GRAPPA-OMERACT PsA core domain set

	With DFP *n* (%)	Without DFP *n* (%)	*p*-value
**Overall**	17 (40)	25 (60)	
**Global disease activity**			
Global disease activity severity (RAPID3)			
Remission	0 (0)	7 (28)	0.028*
Low severity	3 (18)	8 (32)	
Moderate severity	9 (53)	7 (28)	
High severity	5 (29)	3 (12)	
**Physical function**			
Mobility (EQ-5D-3 L)			
I have no problems walking about	7 (42)	17 (68)	0.117
I have some problems walking about	10 (58)	8 (32)	
Able to walk 3 km? (MD-HAQ)			
Without any difficulty	3 (18)	15 (60)	0.026*
With some difficulty	7 (41)	6 (24)	
With much difficulty	1 (6)	0 (0)	
Unable to do so	6 (35)	4 (16)	
**Participation**			
Able to cope with self-care activity? (OT)^+^			
Coping well	15 (88)	21 (88)	0.665
Difficulty with 1–2 tasks	2 (12)	3 (12)	
Able to cope with leisure activity? (OT)^+^			
Yes	13 (86)	19 (95)	0.74
No	1 (14)	2 (5)	
Ability to perform usual activities (For example, work, study, housework, family or leisure activities) (EQ-5D-3 L)^+^			
I have no problems performing my usual activities	9 (53)	14 (56)	0.652
I have some problems performing my usual activities	8 (47)	11 (44)	
Able to participate in recreational activities and sports as you would like? (MD-HAQ)^+^			
Without any difficulty	5 (29)	9 (36)	0.849
With some difficulty	7 (41)	11 (44)	
Unable to do so	5 (29)	5 (20)	
I am unable to carry out my previous work (MFPDI)^+^			
None of time	9 (64)	10 (100)	0.144
On most/every day	2 (14)	0	
On some days	3 (21)	0	
I no longer do all my previous activities (MFPDI)^+^			
None of the time	9 (53)	8 (80)	0.161
On some days	8 (47)	2 (20)	
Living arrangement (MSW)^+^			
Alone	2 (12)	1 (4)	0.385
Family	15 (88)	22 (88)	
Types of domestic tasks engaged in (OT)^+^			
None	0 (0)	1 (4)	0.063
Light	5 (29)	3 (12)	
Moderate	9 (53)	4 (16)	
Heavy	1 (6)	6 (24)	
Not engaging in cardiovascular exercise (PT)	14 (82)	16 (64)	0.300
**Emotional well-being**			
Over the past week, were you able to deal with feelings of anxiety or being nervous? (MD-HAQ)			
Without any difficulty	12 (71)	18 (72)	0.293
With some difficulty	3 (18)	7 (28)	
With much difficulty	1 (4)	0 (0)	
Unable to do so	1 (4)	0 (0)	
Over the past week, were you able to deal with feelings of depression or feeling down? (MD-HAQ)			
Without any difficulty	10 (59)	20 (80)	0.206
With some difficulty	6 (35)	5 (20)	
With much difficulty	1 (6)	0	
Anxiety/Depression (EQ-5D-3 L)			
I am not anxious/depressed	11 (65)	19 (76)	0.576
I am moderately anxious/depressed	5 (29)	6 (24)	
I am extremely anxious/depressed	1 (6)	0 (0)	
**Sleep**			
Able to get a good night’s sleep? (MD-HAQ)			
Without any difficulty	9 (53)	18 (72)	0.424
With some difficulty	6 (35)	6 (24)	
With much difficulty	2 (13)	1 (5)	
**Fatigue**			
Has fatigue been a problem for you over the past month? (OT)^+^			
Yes	7 (44)	4 (17)	0.08
No	9 (56)	20 (83)	

*Statistically significant, ^**+**^Missing data

MD-HAQ multi-dimensional Health Assessment Questionnaire, EQ-5D-3 L EuroQol 5-dimension level-3 questionnaire, RAPID3 Routine Assessment of Patient Index Data-3, MFPDI Manchester Foot Pain and Disability Index, OT Occupational Therapist clinical assessment, MSW Medical Social Worker clinical assessment

Participants were grouped into those with DFP and those without according to the MFPDI score, 17 had DFP and 25 without. The DFP-group compared with those without DFP were: younger (mean (SD) 52-years (16)), had shorter disease duration (median (IQR) 1-year (12)), comparable BMI, but with a higher presence of radiographic damage in the foot (57% versus 13%). The majority of participants with DFP were taking DMARD monotherapy (93%) with concomitant need for NSAIDs (63% compared with 33% in those without DFP). Nearly two-thirds of all participants had a diagnostic referral for hand x-rays (64%, *n* = 27) compared with a much smaller proportion that had been referred for foot x-rays (36%, *n* = 15).

Analyses of global disease measures and disease indices found overall mild-to-moderate disease activity (global pain, Patient and Physician Global Assessment, RAPID3, TSJC-68/66) and burden (EQ-5D-3 L VAS), and low-levels of overall functional impairment (MD-HAQ). All global measures and disease indices were significantly higher in those with DFP (*p* < 0.05) compared to those without DFP, except for ESR (*p* = 0.56) and CRP measures (*p* = 0.78). Participants with DFP had reduced physical function compared to those without DFP (MD-HAQ scores of 0.5 (0.7) vs. 0.2 (0.5)), higher levels of global pain (NRS scores of 4 (2) vs. 2 (4)), higher musculoskeletal disease activity (RAPID3 10.6 (4) vs. 5.6 (5)), and reduced health-related quality of life (EQ-5D-3 L VAS 57.3 (14) vs. 65.8 (27)). Participants with DFP demonstrated significantly higher median (IQR) SJC-66 and TJC-68 scores (8.0 (19.0) and 12.0 (19.0) respectively (*p* < 0.05)) compared to those without DFP, with the talocrural joint and 3rd metatarsophalangeal joint most frequently affected.

Despite the majority of participants reporting to be coping well with their condition (*n* = 38, 90%), undertaking appropriate self-care (*n* = 36, 86%) and having emotional support (*n* = 38, 90%), a high proportion (29%) reported anxiety and depression. High levels of sleep disturbance (34%) and fatigue (24%) were also reported.

The most frequently reported coping strategies were relaxation (*n* = 26, 62%), problem solving (*n* = 11, 26%) and seeking support from social systems (*n* = 11, 26%). 74% (*n* = 26) agreed to receiving information about support groups and helplines for assistance. A lack of disease- and drug-specific knowledge and understanding was reported in nearly two-thirds of participants (64%). Following assessment by all members of the MDT, further management was indicated for medication adherence counselling (48%), occupational therapy (43%), physiotherapy (36%), podiatry (30%) and financial counselling (20%).

72% of participants were overweight or obese (*n* = 30), with a high proportion not engaging in any cardiovascular exercise (*n* = 30, 70%). The majority reported participating in leisure activities identified as sedentary-to-light activity (watching TV, playing electronic devices, sewing (70%)).

Nearly half had current foot pain with 40% reporting DFP (*n* = 17) and there were moderate levels of rearfoot deformity (mean SI rearfoot score 3 (6)). Overall, the most frequent concerns related to walking slowly, difficulty with prolonged standing/walking and undertaking daily routines with more pain. Those with DFP had significantly greater difficulty walking 3 km (76%) than those without (*p* < 0.05). Whilst participants with DFP were twice as likely to work full-time than those without DFP (68%, *n* = 11 compared with 32%, *n* = 8) and spend longer than 3-hours a day on their feet (23% compared with 13%), over one-third (36%) reported being unable to carry out their previous work and compared with none among people without DFP. Rearfoot enthesitis was the most common cause of DFP (67%) with pain lasting longer than 1-year (58%). Most rearfoot enthesitis occurred at the plantar fascia and Achilles entheses, followed by the functional entheses at the peroneal and tibialis posterior tendon sites. Most participants with foot pain (93%) had not sought professional podiatry treatment, even when the pain was disabling.

## Discussion

Disabling foot pain was found in over two-thirds of participants in this PsA-specific study sample in Singapore, which is consistent with previously published results from a UK-based cross-sectional study of self-reported foot pain in PsA [8]. Participants in the current study had shorter disease durations (median 2 years) compared with similar published research from the UK (mean 10 years) and Australia (mean 11 years) [6, 8]. This may be attributed to participants in the earlier stages of disease being considered to benefit the most from holistic care at the specialist outpatient clinic and as a result were more frequently referred. A higher proportion of participants were taking DMARD monotherapy (with biologic use being at 5%, *n* = 2) compared with previously published research from the UK (12%, *n* = 12) and Australia (33%, *n* = 7) [6, 8]. This may be attributed to the high cost of biologic drugs prohibiting their widespread use in Singapore, where public healthcare is subsidised by a system of compulsory savings from payroll deductions with highly variable out-of-pocket payments for services and treatments [49]. Previous research supports the view that those with PsA have lower rates of meeting clinically meaningful response criteria with traditional DMARDs [50], this could partly explain the high frequency and persistence of foot and ankle problems in the study sample. Indeed, inflammation as well as foot pain and related-disability have been observed in a large proportion of people with PsA, despite receiving pharmacological therapy [10, 12, 51–53]. This highlights the potential importance of non-pharmacological management and the role of allied-health professionals, and with limited evidence to date for non-pharmacological interventions in PsA [50] this warrants future research.

This study demonstrates that DFP in PsA negatively impacts on the daily lives of people in Singapore including their walking ability, participation in exercise and leisure activities, and ability to perform household and work tasks. Poorer physical function among individuals with DFP reported in the current study is a finding that is consistent with published PsA-specific research [6, 8, 9]. Previous studies have shown the benefits of physical activity in reducing pain and fatigue, and improving functional capacity and quality of life in PsA [54]. Lower levels of exercise in those with DFP indicate that foot problems and the consequent gait function deficiencies are potential barriers to physical activity and may be contributing to major health issues for people with PsA. This is an important observation from our study as those individuals represent a high-risk group for adverse cardiovascular health. Research in Spondyloarthritis suggests that a potential barrier to physical activity includes personal beliefs related to the onset of pain with exercise [55]. In the current study, the majority (73%) of people with PsA in Singapore reported wanting more information and advice on engaging in physical activity. This suggests that priority of future research should be to identify the potential benefits of a multidisciplinary approach to increasing activity levels that includes; a targeted educational program on the benefits of exercise tailored to people with PsA to improve disease-specific understanding; prescription of exercise therapy; and interventions that address biomechanical abnormalities in the foot to reduce mechanically triggered inflammation and pain in people with PsA with disabling foot problems [56].

Enthesitis is a hallmark and pathognomonic feature of PsA, and has been shown to be largely persistent and non-responsive to standard pharmacological treatment regimens [57], perhaps explaining the higher frequency of rearfoot involvement and foot-related disability in the current study. This finding supports a growing body of evidence in PsA linking foot pain and disease manifestations in the foot with altered patterns of gait and related disability such as reduced walking speed, inconsistent foot loading patterns and increased gait variability [9, 10, 12, 13, 58]. Published studies suggest that rearfoot enthesitis is associated with a higher burden of disease and worse functional outcomes in comparison with those who do not have enthesitis [10]. Further research is required to provide insights into region-specific foot pain, associated movement patterns and potential mechanisms of stress shielding that may help to direct management strategies in early disease to off-load high stress areas and prevent progressive rearfoot disease. Data about musculoskeletal involvement in the foot was obtained through patient-reported descriptions of previous and current foot problems and clinical examination, and the presence of clinical features was not validated by ultrasound imaging. While there is merit in clinic-based research, clinical examination of PsA disease features such as enthesitis has been shown to lack the sensitivity and specificity of ultrasound imaging to detect active disease in PsA [59, 60]. Lack of reliability of clinical foot examinations limits our understanding of psoriatic foot disease, and further research is required to determine whether the common methods used to establish foot involvement in PsA can provide accurate information.

Significant differences were found in global disease activity measures and disease indices between groups with and without DFP. The RAPID3 revealed a significant difference between-groups with greater disease severity (82% reporting moderate-to-high disease activity) in the DFP group compared with those without DFP (40%). There were also significantly fewer in remission and low disease activity (18%) in the DFP group, compared with those without DFP (60%). This confirms the utility of validated, composite tools such as the RAPID3 to better identify those experiencing higher levels of foot disease impact and capture a holistic view of the patient experience and overall disease status in PsA. Although it is acknowledged that composite measures assess multiple dimensions of disease status and that certain domains are not accounted for [39], these study findings suggest that the item inclusion of pain, physical function and patient global assessment in self-report instruments may help to capture foot-specific impact important and relevant to people with PsA. Significant differences between groups were also observed in the SJC-66 and TJC-68. There was a much higher proportion of swollen and tender joints in the foot and ankle in those with DFP compared to those without, which suggests that the inclusion of extended joint counts can potentially identify those with higher levels of foot-disease burden in people living with PsA [61]. These study findings indicate the potential benefit of foot-specific measures and their inclusion within a core set of PsA metrics when making overall treatment decisions. Currently no guidelines exist for the clinical assessment of foot pain and related-disability in PsA. Further research into the development and validation of foot-specific outcome measures in PsA is required in ordered to identify and support of these individuals as well as inform on future podiatry service planning.

Consistent with Australian-based PsA-specific qualitative research that has shown a high foot disease burden and wide-reaching life impact [6], the current study found higher levels of impact among those experiencing DFP including; more severe disease activity, poorer mobility, a higher frequency of fatigue and a greater emotional burden. The various impact domains examined were multifactorial with multidirectional relationships with foot pain and each other, which highlights the importance for clinicians managing foot problems to consider the holistic wellbeing of people with PsA when treating them. This study presents a unique, transdisciplinary, collaborative approach to patient care in PsA in Singapore, with strong incorporation of patient-reported measures, concerns and coping ability in order to capture the patient experience and personal impact – often poorly recognised by health professionals [6].

Effective self-management strategies included positive coping skills, the ability to self-care and readily available social support. It might be typically expected that people with longer disease duration are more familiar with the coping process than those newly diagnosed and thus make better adjustments and accept changes more easily during the disease course [62]. Asian cultural factors such as the Chinese viewing stoicism as a positive coping mechanism (the enduring of pain silently) [63] may explain the high levels of self-reported ability to cope in the current study sample in early disease. This may also have contributed to the under-reporting of disabling foot problems observed in the current study, as evidenced by the low number of referrals to podiatry services, the lower frequency of diagnostic referrals for foot x-rays compared with hand x-rays, and the higher presence of radiographic damage among those with DFP in early disease. People living with PsA may under-report their foot-related disease burden when it is not explicitly described using foot-specific outcome measures such as the MFPDI, which may represent a potential barrier to receiving timely treatment. This suggests that the integration of podiatry within expert-led rheumatology teams may facilitate detection and effective management of foot involvement for improved foot health outcomes in PsA.

The level of anxiety and depression reported in this study is approximately twice the level reported in the general population in Singapore (14% and 15% respectively) [64]. Whilst there are no PsA-specific local data, the current study found 29% (*n* = 12) of participants with self-reported depression and anxiety, with the DFP group reporting higher levels (depression (41%) and anxiety (29%)) compared with the overall PsA sample. This is consistent with previously published findings showing a bidirectional relationship between depressive symptoms and pain in PsA [65].

Limitations of this study include the small sample size leading to low statistical power to show statistical significance across study variables. The study sample of *n* = 42 was small relative to the period of data collection from 2016 to 2022, which was attributed to the sparsity of the OSAC service provision that was limited by high allocation of clinical resources associated with a MDT care model, the low clinic capacity of 6 patients per clinic session, and the clinic having ceased operation due to the Covid-19 pandemic. In addition, although research on the prevalence and incidence of PsA in Asia has been limited, the reported prevalence of PsA seems to be lower in Asian countries compared with Europe and the United States [15, 16]. The small sample size of the current study may be considered to limit transferability of findings to the wider PsA population. Whilst the current study provides new insight into a Southeast-Asian population with PsA-specific foot involvement allowing initial data comparisons with Australian and UK-based studies, a larger population-based sample is required to comprehensively describe the cultural, genetic and environmental differences.

The limitation of secondary data analysis should be acknowledged as the outcome measures were predefined by the original primary research question and hence there was a lack of PsA-specific outcome measures. For example, the dermatological impact on the foot of skin and toenail psoriasis were poorly recorded. Although footwear type and concerns were recorded by the podiatrist, the information did not progress current knowledge beyond previously published Singapore-specific research on footwear characteristics in people with inflammatory arthritis [66]. Results may not be generalisable to all people with PsA in Singapore as participants were referred to the OSAC based on being likely to benefit from MDT care. Whilst the MFPDI is a validated foot-specific outcome measure suitable for epidemiological research in foot pain [24], its level of content validity for use in PsA is unknown. Future work may be indicated to assess the conceptual coverage of items of the MFPDI in its evaluation of foot disease in PsA and cross culturally in Asian populations. No recall period was used in the VAS for foot pain (such as, how severe has your foot pain been over the past week?), which may have influenced the accuracy and reliability of self-reported foot pain severity. Furthermore, findings from this study may be subject to bias as confounding variables were not adjusted for, such as the comorbidity diabetes, which is known to negatively impact on foot health. However, eliminating the impact of co-morbidities comes at the expense of external validity and loss of generalisability in a real-world context.

Despite these limitations, the current study is (to the best of the authors’ knowledge) the first PsA foot-focused study in Singapore and presents a unique, integrated data set on PsA-related foot problems. Future research sampling a larger PsA population across multiple centers in Singapore and across the world is warranted in order to further substantiate these findings, which may help to inform future targeted disease management strategies for improved patient outcomes and experience in PsA, as well as to facilitate future comparative study with other countries on localised disease impact.

## Conclusion

People with DFP in PsA in Singapore experience higher levels of negative impact on their daily lives compared with those without, including significantly more severe global disease activity, poorer physical function, reduced participation in exercise, leisure and work activities, a higher frequency of fatigue and a greater emotional burden. Study findings suggest that the inclusion and utility of foot-specific measures in the clinical assessment of PsA is important in order to identify those with disability and provide appropriate care. Knowledge of the patients’ perception of their level of physical activity and participation, coping ability and emotional wellbeing, should facilitate person-centred care with the potential to improve outcomes in PsA. In the absence of working in a MDT, we recommend the value of comprehensive assessment to capture a holistic view of the multifaceted personal impact in PsA.

## Data Availability

The datasets used and/or analysed during the current study are available from the corresponding author on reasonable request.

## Abbreviations

PsA: Psoriatic Arthritis
DFP: Disabling Foot Pain
MDT: Multidisciplinary team
NSAIDs: Non-Steroidal Anti-Inflammatory Drugs
ESR: Erythrocyte Sedimentation Rate
CRP: C-Reactive Protein
DMARDs: Disease modifying anti-rheumatic drugs
csDMARDs: Conventional synthetic DMARDs
SD: Standard Deviation
IQR: Interquartile Range
TSJC: Tender and Swollen Joint Count
SI: Structural Index
MD-HAQ: Multi-dimensional Health Assessment Questionnaire
RAPID3: Routine Assessment of Patient Index Data-3
EQ-5D-3 L: EuroQoL 5-dimensional level-3 questionnaire
MFPDI: Manchester Foot Pain and Disability Index
VAS: Visual Analogue Scale
NRS: Numerical Rating Scale
IPJ: Inter-phalangeal joint
SD: Standard Deviation
IQR: Interquartile Range
